# Supplementary material for: Sequence-structure-function relationships in class I MHC: A local frustration perspective
Source: PLoS One. 2020 May 18;15(5):e0232849. doi: 10.1371/journal.pone.0232849 (PMC7233585; doi:10.1371/journal.pone.0232849)
Supplement: S1 Table — Clusters are also indicated with numbers (1, 2, or 3). HLA core alleles (taken from Robinson et al. [54]) are shown in bold. (DOCX) [file pone.0232849.s001.docx]

**Supplementary Table 1 – HLA I alleles for which homology models were generated**. Clusters are also indicated with numbers (1, 2, or 3). HLA core alleles (taken from Robinson et al. [1]) are shown in bold.

| **Allele List** | **Cluster** |
| --- | --- |
| **B*07:02**, B*07:03, B*07:04, B*07:05, B*07:06, B*07:07, B*07:09, B*07:10, B*07:11, B*07:14, B*07:15, B*07:16, B*07:17, B*07:18, B*07:20, B*07:26, B*07:31, B*07:32, B*07:33, B*07:36, B*07:38, B*07:40, B*07:41, B*07:42, B*07:44, B*07:46, B*07:47, B*07:48, B*07:50, B*07:53, B*07:57, B*07:58, B*07:59, B*07:61, B*07:65, B*07:66, B*07:69, B*07:77, B*07:86, B*07:92, **B*08:01**, B*08:02, B*08:09, B*08:17, B*08:18, B*08:20, B*08:23, B*08:29, B*08:32, B*08:33, B*08:34, B*08:35, B*08:36, B*08:37, B*08:39, B*08:62, B*13:01, **B*13:02**, B*13:03, B*13:04, B*13:08, B*13:11, B*13:12, B*13:15, B*13:22, B*13:25, B*13:26, B*13:36, B*13:38, B*13:64, B*13:70, B*14:01, B*14:02, B*14:03, B*14:05, B*14:06, B*14:10, B*14:12, **B*15:01**, B*15:02, B*15:03, B*15:04, B*15:05, B*15:07, B*15:08, B*15:09, B*15:10, B*15:11, B*15:12, B*15:13, B*15:14, B*15:15, B*15:16, B*15:17, B*15:18, B*15:19, B*15:20, B*15:21, B*15:23, B*15:24, B*15:25, B*15:27, B*15:28, B*15:29, B*15:31, B*15:32, B*15:33, B*15:34, B*15:35, B*15:37, B*15:42, B*15:44, B*15:45, B*15:46, B*15:48, B*15:50, B*15:51, B*15:52, B*15:54, B*15:55, B*15:56, B*15:58, B*15:63, B*15:65, B*15:66, B*15:68, B*15:70, B*15:71, B*15:72, B*15:73, B*15:77, B*15:81, B*15:82, B*15:83, B*15:84, B*15:86, B*15:89, B*15:92, B*15:93, B*15:95, **B*18:01**, B*18:02, B*18:03, B*18:06, B*18:09, B*18:13, B*18:14, B*18:19, B*18:20, B*18:22, B*18:25, B*18:26, B*18:28, B*18:32, B*18:33, B*18:40, B*18:96, B*27:01, B*27:02, B*27:03, B*27:04, **B*27:05**, B*27:06, B*27:07, B*27:08, B*27:09, B*27:10, B*27:11, B*27:12, B*27:13, B*27:14, B*27:15, B*27:17, B*27:19, B*27:20, B*27:24, B*27:25, B*27:30, B*27:32, B*27:35, B*27:38, B*27:45, B*27:47, B*27:51, B*27:52, B*27:67, B*27:68, B*27:69, B*27:96, **B*35:01**, B*35:02, B*35:03, B*35:04, B*35:05, B*35:06, B*35:07, B*35:08, B*35:09, B*35:10, B*35:11, B*35:12, B*35:13, B*35:14, B*35:15, B*35:16, B*35:19, B*35:20, B*35:21, B*35:22, B*35:23, B*35:26, B*35:27, B*35:28, B*35:30, B*35:31, B*35:33, B*35:34, B*35:35, B*35:36, B*35:38, B*35:39, B*35:41, B*35:42, B*35:43, B*35:44, B*35:46, B*35:47, B*35:49, B*35:50, B*35:51, B*35:52, B*35:55, B*35:57, B*35:60, B*35:63, B*35:67, B*35:70, B*35:76, B*35:77, B*35:78, B*35:79, B*35:81, B*35:82, B*35:83, B*35:87, B*35:90, B*35:92, B*35:93, B*35:94, B*35:95, **B*37:01**, B*37:02, B*37:04, B*37:07, B*37:09, B*37:10, B*37:23, **B*38:01**, B*38:02, B*38:05, B*38:06, B*38:09, B*38:11, B*38:12, B*38:14, B*38:17, B*38:18, B*38:28, B*38:46, B*38:49, B*38:50, B*39:01, B*39:02, B*39:03, B*39:04, B*39:05, B*39:06, B*39:07, B*39:08, B*39:09, B*39:10, B*39:11, B*39:12, B*39:13, B*39:14, B*39:15, B*39:20, B*39:22, B*39:23, B*39:24, B*39:28, B*39:34, B*39:36, B*39:37, B*39:41, B*39:42, B*39:44, B*39:45, B*39:46, B*39:47, B*39:49, B*39:50, B*39:58, B*39:59, B*39:60, B*39:85, B*39:86, B*40:0, **B*40:01**, B*40:02, B*40:03, B*40:04, B*40:05, B*40:06, B*40:07, B*40:08, B*40:10, B*40:11, B*40:12, B*40:13, B*40:14, B*40:16, B*40:19, B*40:23, B*40:26, B*40:29, B*40:30, B*40:31, B*40:32, B*40:37, B*40:39, B*40:40, B*40:42, B*40:43, B*40:48, B*40:49, B*40:50, B*40:51, B*40:52, B*40:53, B*40:54, B*40:55, B*40:56, B*40:57, B*40:58, B*40:61, B*40:63, B*40:66, B*40:67, B*40:68, B*40:70, B*40:72, B*40:73, B*40:77, B*40:79, B*40:81, B*40:82, B*40:85, B*40:86, B*40:88, B*40:89, B*40:90, B*40:91, B*40:94, B*40:97, B*40:98, B*40:99, B*41:01, B*41:02, B*41:07, B*42:0, B*42:01, B*42:02, B*42:06, B*42:08, B*42:13, **B*44:02**, B*44:03, B*44:04, B*44:05, B*44:06, B*44:07, B*44:08, B*44:09, B*44:10, B*44:11, B*44:12, B*44:13, B*44:14, B*44:16, B*44:17, B*44:18, B*44:20, B*44:21, B*44:24, B*44:26, B*44:29, B*44:30, B*44:31, B*44:32, B*44:39, B*44:46, B*44:49, B*44:50, B*44:55, B*44:57, B*44:59, B*44:64, B*44:65, B*44:66, B*44:77, B*44:81, B*44:83, B*45:0, **B*45:01**, B*45:02, B*45:04, B*45:07, B*45:09, B*47:01, B*47:02, B*47:03, B*47:04, B*48:01, B*48:02, B*48:03, B*48:04, B*48:05, B*48:06, B*48:07, B*48:08, B*48:09, B*48:10, B*48:12, B*48:13, B*48:23, B*48:34, B*49:01, B*49:02, B*49:05, B*49:07, B*50:01, B*50:02, B*50:04, B*50:37, **B*51:01**, B*51:02, B*51:03, B*51:04, B*51:05, B*51:07, B*51:08, B*51:14, B*51:16, B*51:17, B*51:19, B*51:20, B*51:22, B*51:29, B*51:30, B*51:31, B*51:32, B*51:35, B*51:36, B*51:37, B*51:39, B*51:40, B*51:42, B*51:48, B*51:50, B*51:51, B*51:56, B*51:57, B*51:59, B*51:64, B*51:65, B*51:79, B*51:83, B*51:89, B*51:90, B*51:96, B*52:01, B*52:03, B*52:04, B*52:05, B*52:06, B*52:07, B*52:08, B*52:11, B*52:12, B*52:14, B*52:20, B*52:29, B*52:34, B*52:35, B*52:36, B*53:0, B*53:01, B*53:07, B*53:10, B*53:11, B*53:14, B*54:0, **B*54:01**, B*54:04, B*54:06, B*54:07, B*54:14, B*54:15, B*54:17, B*54:18, B*54:21, B*54:22, B*54:31, B*54:32, B*55:0, B*55:01, B*55:02, B*55:03, B*55:04, B*55:05, B*55:07, B*55:10, B*55:11, B*55:12, B*55:18, B*55:22, B*55:24, B*55:26, B*55:28, B*55:30, B*55:32, B*55:37, B*55:41, B*55:42, B*55:43, B*55:48, B*55:57, B*55:61, B*55:62, B*55:63, B*56:01, B*56:02, B*56:03, B*56:04, B*56:06, B*56:07, B*56:10, B*56:11, B*56:15, B*56:18, B*56:21, B*56:24, B*56:27, **B*57:01**, B*57:02, B*57:03, B*57:05, B*57:06, B*57:11, B*57:29, B*58:01, B*58:02, B*58:09, B*58:11, B*58:15, B*58:19, B*58:20, B*58:26, B*58:29, B*58:45, B*58:49, B*58:50, B*58:51, B*58:52, B*58:54, B*58:55, B*58:56, B*58:57, B*58:58, B*59:01, B*59:02, B*59:03, B*59:04, B*59:05, B*67:0, B*67:01, B*67:04, **B*73:01**, B*78:01, B*78:02, B*78:04, B*78:05, B*78:06, B*81:0, B*81:01, B*81:02, B*81:03, B*81:05, B*82:02, B*83:01 | 1 |
| B*46:0, B*46:01, B*46:03, B*46:04, B*46:05, B*46:08, B*46:11, B*46:12, B*46:14, B*46:16, B*46:18, B*46:19, B*46:23, B*46:24, B*56:14, B*67:02, **C*01:02**, C*01:04, C*01:05, C*01:06, C*01:08, C*01:11, C*01:12, C*01:13, C*01:14, C*01:17, C*01:21, C*01:22, C*01:25, C*01:27, C*01:30, C*01:38, C*01:39, C*01:40, **C*02:02**, C*02:03, C*02:07, C*02:08, C*02:10, C*02:11, C*02:12, C*02:13, C*02:16, C*02:17, C*02:20, C*02:21, C*02:23, C*02:29, C*02:32, C*02:35, C*02:69, C*02:71, **C*03:02**, C*03:03, C*03:04, C*03:05, C*03:06, C*03:08, C*03:09, C*03:10, C*03:13, C*03:14, C*03:16, C*03:19, C*03:23, C*03:26, C*03:28, C*03:29, C*03:30, C*03:32, C*03:34, C*03:35, C*03:36, C*03:40, C*03:41, C*03:42, C*03:44, C*03:46, C*03:47, C*03:49, C*03:51, C*03:53, C*03:55, C*03:58, C*03:61, C*03:62, C*03:74, C*03:75, C*03:81, C*03:82, C*03:85, C*03:93, **C*04:01**, C*04:03, C*04:05, C*04:06, C*04:08, C*04:10, C*04:11, C*04:14, C*04:19, C*04:23, C*04:26, C*04:27, C*04:28, C*04:29, C*04:30, C*04:33, C*04:36, C*04:38, C*04:40, C*04:41, C*04:42, C*04:49, C*04:56, C*04:63, C*04:65, C*04:70, **C*05:01**, C*05:03, C*05:08, C*05:09, C*05:10, C*05:13, C*05:14, C*05:19, C*05:23, C*05:25, C*05:26, C*05:27, C*05:35, C*05:37, C*05:45, **C*06:02**, C*06:06, C*06:07, C*06:08, C*06:09, C*06:12, C*06:13, C*06:14, C*06:23, C*06:24, C*06:27, C*06:29, C*06:30, C*06:38, C*06:39, C*06:45, C*06:83, C*06:99, **C*07:01**, C*07:02, **C*07:04**, C*07:05, C*07:06, C*07:07, C*07:10, C*07:11, C*07:12, C*07:13, C*07:14, C*07:15, C*07:16, C*07:18, C*07:19, C*07:20, C*07:22, C*07:24, C*07:25, C*07:26, C*07:28, C*07:29, C*07:30, C*07:35, C*07:36, C*07:37, C*07:39, C*07:40, C*07:41, C*07:42, C*07:45, C*07:46, C*07:49, C*07:50, C*07:51, C*07:52, C*07:56, C*07:57, C*07:59, C*07:60, C*07:63, C*07:64, C*07:66, C*07:67, C*07:68, C*07:70, C*07:72, C*07:74, C*07:75, C*07:80, C*07:81, C*07:89, C*07:93, **C*08:01**, C*08:02, C*08:03, C*08:04, C*08:05, C*08:06, C*08:08, C*08:10, C*08:11, C*08:12, C*08:13, C*08:15, C*08:17, C*08:20, C*08:21, C*08:22, C*08:24, C*08:25, C*08:27, C*08:28, C*08:30, C*08:31, C*08:32, C*08:34, C*08:40, C*08:78, C*08:95, C*08:96, C*08:97, C*08:98, C*08:99, **C*12:02**, C*12:03, C*12:04, C*12:05, C*12:08, C*12:09, C*12:11, C*12:12, C*12:13, C*12:16, C*12:19, C*12:20, C*12:22, C*12:23, C*12:24, **C*14:02**, C*14:03, C*14:04, C*14:05, C*14:06, C*14:09, C*14:11, C*14:20, **C*15:02**, C*15:03, C*15:04, C*15:05, C*15:06, C*15:07, C*15:08, C*15:09, C*15:11, C*15:13, C*15:15, C*15:16, C*15:17, C*15:18, C*15:22, C*15:24, C*15:25, C*15:26, C*15:29, **C*16:01**, C*16:02, C*16:04, C*16:08, C*16:09, C*16:18, C*16:25, C*16:63, **C*17:01**, C*17:02, C*17:03, C*17:04, C*17:05, C*18:01, C*18:02 | 2 |
| **A*01:01**, A*01:02, A*01:03, A*01:06, A*01:09, A*01:14, A*01:17, A*01:20, A*01:23, A*01:25, A*01:32, A*01:37, A*01:43, A*01:45, **A*02:01**, A*02:02, A*02:03, A*02:04, **A*02:05**, A*02:06, A*02:07, A*02:08, A*02:09, A*02:10, A*02:11, A*02:12, A*02:13, A*02:14, A*02:15, A*02:16, A*02:17, A*02:18, A*02:19, A*02:20, A*02:21, A*02:22, A*02:23, A*02:24, A*02:25, A*02:26, A*02:27, A*02:28, A*02:29, A*02:30, A*02:33, A*02:34, A*02:36, A*02:38, A*02:39, A*02:41, A*02:42, A*02:44, A*02:47, A*02:48, A*02:49, A*02:51, A*02:52, A*02:54, A*02:57, A*02:58, A*02:59, A*02:60, A*02:61, A*02:64, A*02:65, A*02:66, A*02:67, A*02:68, A*02:69, A*02:70, A*02:71, A*02:72, A*02:73, A*02:75, A*02:77, A*02:79, A*02:80, A*02:81, A*02:85, A*02:87, A*02:89, A*02:90, A*02:95, A*02:96, **A*03:01**, A*03:02, A*03:04, A*03:06, A*03:08, A*03:09, A*03:10, A*03:12, A*03:18, A*03:20, A*03:25, A*03:26, A*03:35, A*03:37, A*03:39, A*03:42, A*03:43, A*03:45, A*03:47, A*03:53, A*03:55, A*03:56, A*03:57, A*03:60, A*03:62, A*03:64, A*03:71, A*03:72, A*03:77, A*03:78, A*03:82, **A*11:01**, A*11:02, A*11:03, A*11:04, A*11:05, A*11:07, A*11:08, A*11:09, A*11:10, A*11:12, A*11:13, A*11:19, A*11:25, A*11:29, A*11:35, A*11:36, A*11:38, A*11:41, A*11:48, A*11:49, A*11:55, A*11:56, A*11:57, A*11:60, A*11:61, A*11:62, A*23:01, A*23:04, A*23:05, A*23:06, A*23:09, A*23:15, A*23:16, A*23:17, A*23:18, A*23:20, A*23:26, **A*24:02**, A*24:03, A*24:04, A*24:07, A*24:08, A*24:10, A*24:11, A*24:12, A*24:13, A*24:14, A*24:17, A*24:20, A*24:22, A*24:23, A*24:25, A*24:26, A*24:28, A*24:30, A*24:31, A*24:32, A*24:33, A*24:35, A*24:37, A*24:39, A*24:43, A*24:46, A*24:47, A*24:49, A*24:50, A*24:52, A*24:53, A*24:54, A*24:56, A*24:58, A*24:59, A*24:61, A*24:62, A*24:63, A*24:68, A*24:71, A*24:75, A*24:76, A*24:78, A*24:79, A*24:80, A*24:81, A*24:85, A*24:87, A*24:88, A*24:89, A*24:91, A*24:92, A*24:93, A*24:95, A*24:96, A*24:98, A*24:99, A*25:01, A*25:02, A*25:04, A*25:05, A*25:07, A*25:09, A*25:11, **A*26:01**, A*26:02, A*26:03, A*26:04, A*26:05, A*26:06, A*26:07, A*26:08, A*26:09, A*26:10, A*26:12, A*26:14, A*26:15, A*26:17, A*26:18, A*26:20, A*26:21, A*26:22, A*26:23, A*26:24, A*26:26, A*26:27, A*26:31, A*26:33, A*26:36, A*26:37, A*26:39, A*26:42, A*26:47, A*26:50, A*26:74, A*26:98, A*29:01, **A*29:02**, A*29:03, A*29:04, A*29:09, A*29:11, A*29:12, A*29:18, A*29:22, A*29:39, A*29:40, A*29:46, A*29:56, A*29:57, A*29:58, **A*30:01**, A*30:02, A*30:03, A*30:04, A*30:07, A*30:08, A*30:09, A*30:10, A*30:16, A*30:18, A*30:20, A*30:23, A*30:24, A*30:25, A*30:28, A*30:31, A*30:33, A*30:38, A*30:39, A*30:79, A*31:01, A*31:02, A*31:04, A*31:05, A*31:06, A*31:08, A*31:09, A*31:10, A*31:11, A*31:12, A*31:13, A*31:15, A*31:16, A*31:17, A*31:18, A*31:20, A*31:22, A*31:23, A*31:24, A*31:29, A*31:31, A*31:32, A*31:33, A*31:34, A*31:36, A*31:46, A*31:56, A*31:65, A*31:74, A*31:81, A*31:82, A*32:01, A*32:02, A*32:03, A*32:04, A*32:05, A*32:06, A*32:07, A*32:08, A*32:15, A*32:17, A*32:22, A*32:24, A*33:01, A*33:03, A*33:05, A*33:07, A*33:08, A*33:09, A*33:11, A*33:15, A*33:16, A*33:17, A*33:19, A*33:25, A*33:31, A*33:49, A*33:76, A*33:77, A*33:78, A*33:79, A*33:81, A*33:82, A*33:83, A*33:84, A*33:85, A*34:01, A*34:02, A*34:05, A*34:06, A*36:01, A*36:03, A*36:04, A*36:05, A*43:01, A*66:01, A*66:02, A*66:07, A*66:08, A*66:13, A*66:14, A*68:01, A*68:02, A*68:03, A*68:05, A*68:06, A*68:07, A*68:08, A*68:10, A*68:12, A*68:15, A*68:16, A*68:17, A*68:19, A*68:20, A*68:22, A*68:23, A*68:24, A*68:25, A*68:31, A*68:33, A*68:35, A*68:36, A*68:38, A*68:43, A*68:54, A*68:71, A*68:96, A*69:01, A*74:0, **A*74:01**, A*74:02, A*74:03, A*74:06, A*74:07, A*74:09, A*74:11, **A*80:01**, A*80:02 | 3 |

1. Robinson J, Guethlein LA, Cereb N, Yang SY, Norman PJ, Marsh SGE, et al. Distinguishing functional polymorphism from random variation in the sequences of >10,000 HLA-A, -B and -C alleles. Keating BJ, editor. PLoS Genet. 2017;13: e1006862. doi:10.1371/journal.pgen.1006862
