# Supplementary material for: Sequence-structure-function relationships in class I MHC: A local frustration perspective
Source: PLoS One. 2020 May 18;15(5):e0232849. doi: 10.1371/journal.pone.0232849 (PMC7233585; doi:10.1371/journal.pone.0232849)
Supplement: S2 Table — The rows are ordered according to increasing rvET. (DOCX) [file pone.0232849.s002.docx]

**Supplementary Table 2 – Median SRFI and rvET values of selected positions in HLA I peptide binding groove and α-3 domain as shown in Fig 5 and Fig 7**. The rows are ordered according to increasing rvET.

| **Position** | **Median SRFI** | **rvET** |
| --- | --- | --- |
| 101 | 1.90 | 1.68 |
| 164 | 1.86 | 2.16 |
| 8 | 1.48 | 5.57 |
| 7 | 0.90 | 6.36 |
| 36 | 1.48 | 6.77 |
| 27 | 1.00 | 7.43 |
| 146 | -1.36 | 7.54 |
| 25 | 1.57 | 7.74 |
| 155 | -0.81 | 8.38 |
| 115 | -0.87 | 9.77 |
| 68 | -1.73 | 9.91 |
| 96 | -0.70 | 10.31 |
| 61 | -1.24 | 10.33 |
| 58 | -2.04 | 11.29 |
| 124 | 1.50 | 11.9 |
| 141 | -0.76 | 12.7 |
| 34 | 1.72 | 14.75 |
| 98 | 1.19 | 17.1 |
| 5 | 0.94 | 18.32 |
| 84 | -0.77 | 18.9 |
| 144 | -0.86 | 28.85 |
| 145 | -0.48 | 56.09 |
| 80 | -0.74 | 85.8 |
| 99 | 0.828 | 107.94 |
| 77 | -0.78 | 121.41 |
| 114 | -0.78 | 153.83 |
| **Position** | **Median SRFI** | **rvET** |
| 259 | 1.80 | 1.0 |
| 257 | 1.03 | 1.5 |
| 204 | 0.99 | 1.54 |
| 266 | 1.15 | 1.63 |
| 247 | 1.40 | 1.63 |
| 243 | -1.13 | 1.68 |
| 249 | 1.29 | 1.72 |
| 212 | -1.06 | 2.12 |
| 198 | -1.30 | 2.17 |
| 208 | 1.36 | 2.27 |
| 201 | 1.22 | 2.37 |
| 227 | -1.94 | 2.52 |
| 230 | 1.79 | 2.6 |
| 210 | -1.25 | 2.6 |
| 223 | -1.92 | 2.87 |
| 213 | 1.41 | 2.92 |
| 215 | 1.40 | 3.1 |
| 254 | -1.20 | 3.22 |
| 256 | 1.75 | 3.58 |
| 203 | 1.56 | 3.74 |
| 241 | 1.10 | 3.78 |
| 196 | -1.16 | 3.95 |
| 261 | 1.47 | 4.27 |
| 248 | 1.30 | 4.74 |
| 232 | 0.89 | 4.9 |
| 231 | 1.98 | 5.07 |
| 189 | 1.33 | 5.07 |
| 229 | -1.14 | 5.41 |
| 222 | -1.67 | 6.74 |
| 183 | -1.13 | 6.92 |
